# Identification and Characterization of Lipase Activity and Immunogenicity of LipL from *Mycobacterium tuberculosis*

**DOI:** 10.1371/journal.pone.0138151

**Published:** 2015-09-23

**Authors:** Jun Cao, Guanghui Dang, Huafang Li, Tiantian Li, Zhiguo Yue, Na Li, Yajun Liu, Siguo Liu, Liping Chen

**Affiliations:** 1 Division of Bacterial Diseases, State Key Laboratory of Veterinary Biotechnology, Harbin Veterinary Research Institute, Chinese Academy of Agricultural Sciences, Harbin, PR China; 2 Heilongjiang Provincial Hospital for Prevention and Treatment of Tuberculosis, Harbin, PR China; Bose Institute, INDIA

## Abstract

Lipids and lipid-metabolizing esterases/lipases are highly important for the mycobacterial life cycle and, possibly, for mycobacterial virulence. In this study, we expressed 10 members of the Lip family of *Mycobacterium tuberculosis*. Among the 10 proteins, LipL displayed a significantly high enzymatic activity for the hydrolysis of long-chain lipids. The optimal temperature for the lipase activity of LipL was demonstrated to be 37°C, and the optimal pH was 8.0. The lipase active center was not the conserved motif G-x-S-x-G, but rather the S-x-x-K and GGG motifs, and the key catalytic amino acid residues were identified as G50, S88, and K91, as demonstrated through site-directed mutagenesis experiments. A three-dimensional modeling structure of LipL was constructed, which showed that the GGG motif was located in the surface of a pocket structure. Furthermore, the subcellular localization of LipL was demonstrated to be on the mycobacterial surface by Western blot analysis. Our results revealed that the LipL protein could induce a strong humoral immune response in humans and activate a CD8^+^ T cell-mediated response in mice. Overall, our study identified and characterized a novel lipase denoted LipL from *M*. *tuberculosis*, and demonstrated that LipL functions as an immunogen that activates both humoral and cell-mediated responses.

## Introduction

Tuberculosis (TB) is an infectious disease caused by bacteria in the *Mycobacterium tuberculosis* complex, which includes *M*. *tuberculosis*, *M*. *bovis*, *M*. *africanum*, *M*. *microti*, and *M*. *canettii* [1, 2]. TB remains one of the world’s deadliest communicable diseases. In 2013, an estimated 9.0 million people developed TB, and 1.5 million died from the disease [3]. The emergence of multi-drug resistant (MDR) and extensively drug resistant (XDR) mycobacterial strains further complicates the prevention and control of TB. Approximately 480,000 people worldwide developed MDR-TB in 2013. It is estimated that approximately 9.0% of the MDR-TB cases had XDR-TB; which underscores the challenges of preventing or treating TB [3]. Therefore, the development of effective vaccines and screening of new drugs against TB is of utmost importance. The elaboration of the pathogenic molecular mechanisms of TB infection may provide valuable insights for vaccine development and new drug screening. Although much has been learned about the pathogen *M*. *tuberculosis*, many aspects of the molecular pathogenicity of *M*. *tuberculosis* remain to be elucidated.

Lipids and esterases/lipases associated with lipid metabolism are very important in the mycobacterial life cycle. Eight percent of the *M*. *tuberculosis* genome encodes products that participate in lipid metabolism, and approximately 30–40% of the dry weight of *M*. *tuberculosis* corresponds to lipids [4, 5]. *M*. *tuberculosis* is a slow-growing intracellular pathogen, and the special pathological characteristic of its infection is granuloma. During the formation phase of granuloma, *M*. *tuberculosis* goes into a dormant state, in which the bacteria accumulate lipids in the form of lipid inclusion bodies (LIBs) [6, 7]. Most of these lipids consists of tri-acylglycerols (TAG) and may originate from host lipid degradation and/or fatty acid absorption [7, 8]. It has been reported that *M*. *tuberculosis* in the center of granulomas can also accumulate lipids from the degradation of immune cells [6, 9]. Furthermore, *M*. *tuberculosis* can convert macrophages, which are colonized by *M*. *tuberculosis*, to exhibit a foamy phenotype and accumulate LIBs through the foamy macrophages [10]. On the one hand, the lipids provide energy to *M*. *tuberculosis*, and on the other hand, these lipids may also provide materials for the synthesis of *M*. *tuberculosis* cell membrane and cell well lipids [11].

Because lipids are important for the survival and pathogenicity of *M*. *tuberculosis*, the esterases/lipases associated with lipid metabolism are considered critical virulence factors for *M*. *tuberculosis* [12]. Lipases catalyze the hydrolysis of ester bonds in long-chain acylglycerols to release fatty acids and glycerol. During infection, *M*. *tuberculosis* also relies on its lipases to hydrolyze host cell lipids to release fatty acids, which serve as its energy source [13, 14].

Lipases differ from esterases due to their ability to hydrolyze substrates with long-chain acylglycerols at the oil-water interface, whereas esterases can only hydrolyze substrates with short-chain acylglycerols [15, 16]. The *M*. *tuberculosis* genome contains a markedly high number of lipolytic enzymes, of which the 21 members of a family called Lip (A to W, except K and S) have been annotated as putative esterases or lipases based on the presence of the G-x-S-x-G motif, which is characteristic of the α/β hydrolase-fold family [17, 18]. In this study, the *rv1497* gene from *M*. *tuberculosis* H37Rv encoding LipL, which was previously annotated as a putative lipase, was overexpressed in *M*. *smegmatis*. We identified and characterized the lipase activity by using the purified LipL protein. Moreover, purified LipL was inoculated in BALB/c mice to evaluate its immunogenicity.

## Materials and Methods

### Ethics statement

Blood samples were obtained from TB patients who were admitted to the Heilongjiang Provincial Hospital for Prevention and Treatment of Tuberculosis, Harbin, China. The sera from healthy adult donors were from Harbin, China. Informed written consent was obtained from all participants. This project was approved by the Ethics Committee of Heilongjiang Provincial Hospital for Prevention and Treatment of Tuberculosis.

The mice used in this study were purchased from Vital River, Beijing, China. The animal experiments were performed in accordance with animal ethics guidelines and approved protocols. The animal experiment was approved by the Animal Ethics Committee of Harbin Veterinary Research Institute of Chinese Academy of Agricultural Sciences. The study approval number was SQ20142049. All the experiments were designed to minimize the numbers of animals used, and every effort was made to minimize pain and distress to the animals.

### Bacterial strains and culture condition

*Escherichia coli* DH5α and BL21 (DE3) (Novagen, Darmstadt, Germany) strains were used as host strains for cloning and expression experiments. *E*. *coli* were grown on Luria-Bertani (LB) broth or agar containing appropriate antibiotics, ampicillin or kanamycin, at 50 μg/ml; Liquid *M*. *smegmatis* cultures were grown in Middlebrook 7H9 medium (BD Biosciences) supplemented with 10% oleic acid/albumin/dextrose/catalase enrichment (10% OADC, BD Biosciences), 0.05% Tween 80 (Amresco), and 0.2% glycerol, containing kanamycin, at 25 μg/ml. Transformants were selected on Middlebrook 7H10 solid media supplemented with 25 μg/ml kanamycin when necessary. Plates were incubated at 37°C for 3 to 4 days for *M*. *smegmatis*.

### Gene amplification and plasmids construction

The parent vector pMV261 (kind gift from Lu Yu) was a promoterless *Escherichia coli*-mycobacteria shuttle plasmid. A pair of primers named “AI-PF” and “AI-PR” was designed (Table 1) according to the sequence from NCBI and used to amplify the acetamidase promoter from *M*. *smegmatis* mc^2^155 genome DNA. The pMV261 inserted with the acetamidase promoter is an acetamide-inducible vector named pAI. Then “Linker-N” (Table 1) containing His-tag was added to vector pAI to generate pAIN vector whose His-tag was at the N-terminal.

**Table 1:** Primers used in this study.

Primer	Sequence (5’→3’)
Primers for vector	
AI-PF	CTAGGTACCAGTGACGCGGTCTCAAGCG
AI-PR	CTAGGATCCAAAACTACCTCGGGCATGTGG
Linker-NF	GATCCTCACCACCACCACCACCACACTAGTCAGCTGCAGAATTCATATGCATCGATGGTT
Linker-NR	AACCATCGATGCATATGAATTCTGCAGCTGACTAGTGTGGTGGTGGTGGTGGTGAG
Primers for mutations	
G49-F	GGTTCGCTGGCGGAGCGCTGGCGGTGT
G49-R	TCCGCCAGCGAACCGGCGCCCCGGAAA
G50-F	GGTTCGGTGCCGGAGCGCTGGCGGTGT
G50-R	TCCGGCACCGAACCGGCGCCCCGGAAA
G51-F	GGTTCGGTGGCGCAGCGCTGGCGGTGTA
G51-R	TGCGCCACCGAACCGGCGCCCCGGAAA
S88-F	GATGGTGTTCGCGGCGACCAAGGGCA
S88-R	TCGCCGCGAACACCATCGGCGCGGAAT
K91-F	GCGGGCATGACGGCCACGGTCATCCA
K91-R	GCCGTCATGCCCGCGGTCGCCGAGAA
S361-F	GCTTGGGCGGCGCGATCGGCTGGACA
S361-R	TCCAGCCGATCGCGCCGCCCAAG

The primers (Table 2) were designed to amplify the 10 Lip family genes of *M*. *tuberculosis*. PrimeSTAR Max DNA polymerase was used for PCR amplification. PCR involved pre-denaturation at 95°C for 5 min, 30 cycles of denaturation at 98°C for 10 sec, annealing at 60°C for 5 sec, elongation at 72°C for 8 sec, and a post-elongation at 72°C for 1 min. The 10 Lip family genes were then cloned into the pAIN vector.

**Table 2:** Primers used for PCR of 10 Lip family genes from *M*. *tuberculosis*.

Gene	Primer direction	Primer sequence (5’→3’)	Amplicon Size(bp)
*lipI*	Sense	CCGGAATTCGCCCAGTTTGGACAACACCGCC	958
*(rv1400c)*	Antisense	CCGGTTAACTCCGTGTAGCACAACCCGTAGCG	
*lipL*	Sense	CCGGAATTCGATGGTTGACACCGGAGTCGATCA	1285
*(rv1497)*	Antisense	CCGGTTAACGCCCGCCGCGGCTCC	
*lipM*	Sense	CCGGAATTCGGGCGCTCCTCGACTCATCCA	1291
*(rv2284)*	Antisense	CCGGTTAACTGGGATCGCAACCGCGGGG	
*lipN*	Sense	CCGGAATTCGACCAAGAGTCTGCCAGGTGTG	1126
*(rv2970c)*	Antisense	CCGGTTAACAACCCGGCTAAGGTGCGCG	
*lipQ*	Sense	CCGGAATTCGCACATCGCCAGCGTGACTTCG	1261
*(rv2485c)*	Antisense	CCGGTTAACGCTGGCCGGAGGTGACGACA	
*lipT*	Sense	CCGGAATTCGGCCCTGGAGTCGGCTAC	1531
*(rv2045c)*	Antisense	CCGGTTAACATTCGCCAGCGAAAACCCGTC	
*lipU*	Sense	CCGGAATTCGGCGGTCCGGCCGGTGCTA	889
*(rv1076)*	Antisense	CCGGTTAACCCCGGTGGCCTCGCGGATGTA	
*lipV*	Sense	CCGGAATTCGCCCGAAATCCCCATCGCC	670
*(rv3203)*	Antisense	CCGGTTAACGCGCGGTCCCAGTCGACT	
*lipY*	Sense	CGCCATATGTGTCTTATGTTGTTGCGTTGCC	1309
*(rv3097c)*	Antisense	CCGGTTAACGGCGGCGATACCGAGTTG	
*lipZ*	Sense	CCGGAATTCGACATCACCGAGTGTCCGGGAG	862
*(rv1834)*	Antisense	CCGGTTAACGTCGACGAGCAGCGATAGCGC	

The restriction sites integrated into the sequences are underlined.

The template plasmid pAIN-lipL and the complementary mutagenic oligonucleotide pairs (Table 1) were used for PCR amplification to introduce the G49A, G50A, G51A, S88A, K91A, and S361A substitutions. The PCR products were digested with *DpnI* to damage the methylated template plasmid. The resultant mutant plasmids were transformed into *E*. *coli* DH5α cells, and all of the substitutions were confirmed by Sanger sequencing.

### Expression and purification of Lip family proteins from *M*. *tuberculosis*

The *M*. *smegmatis* mc^2^155 cells were transformed with the positive recombinant plasmids by electroporation and incubated on 7H10 agar plates containing 25 μg/ml kanamycin. After being incubated for 3 days at 37°C, single colonies were selected and grown in 5 ml of 7H9 broth with 0.05% Tween 80 and 25 μg/ml kanamycin for 3 days. Expression of His-tagged recombinant proteins in *M*. *smegmatis* was performed in 7H9 medium supplemented with 10% OADC, 0.05% Tween 80 and 0.2% glycerol, containing 25 μg/ml kanamycin. The culturing condition was 37°C at a shaking speed of 160 rpm. Acetamide (Sigma-Aldrich) was added to final concentration of 0.2% (w/v) when *M*. *smegmatis* were grown to an optical density at 600 nm (OD_600_) of ~ 2.0 for the expression of His-tagged recombinant proteins and grown for another 20 hours. Cells were harvested by centrifugation at 5,000 rpm for 10 min at 4°C. The cell pellets were washed twice and then re-suspended in ice-cold washing buffer (20 mM Tris-HCl, 150 mM NaCl, pH 8.0) and then passed through a high-pressure cell disruptor at 4°C (model Tso.75, Constant Systems, UK). The cell debris was removed by centrifugation at 15,000 rpm for 30 min at 4°C. The supernatant was loaded onto a Ni^2+^ Sepharose 6 Fast Flow (GE Healthcare, USA) equilibrated with buffer A (20 mM Tris-HCl, 150 mM NaCl, 10 mM imidazole, pH 8.0) after being passed through a filter membrane (Millipore, 0.22 μm, USA). The column was washed with 10 column volumes of buffer A and an imidazole concentration gradient (20, 40, 60, 80, 100, and 200 mM) buffer. Proteins were eluted with elution buffer (buffer A containing 500 mM imidazole and 20% glycerol). The purified protein was then further purified using anion exchange chromatography RESOURCE^TM^ Q column (1 ml) (GE Healthcare, USA), and chromatography Superdex 75 (GE Healthcare, USA). The collected protein samples were analyzed by 12% sodium dodecyl sulfate polyacrylamide gel electrophoresis (SDS-PAGE) and concentration was determined by using a Pierce BCA protein assay kit (Pierce, USA).

### Lipase assay

Lipase activity was assayed by measuring the amount of *p*-nitrophenol (*p*-NP) released from *p*-NP ester substrate with varying lengths of fatty acids [16]. These substrates included *p*-NP acetate (C_2_), *p*-NP butyrate (C_4_), *p*-NP caproate (C_6_), *p*-NP caprylate (C_8_), *p*-NP laurate (C_12_), *p*-NP myristate (C_14_), *p*-NP palmitate (C_16_), and *p*-NP stearate (C_18_). The *p*-NP esters were dissolved in isopropanol at a concentration of 10 mM. The total lipase activity was assayed using the cell lysate supernatants of 10 recombinant *M*. *smegmatis*. The standard lipase activity assays were performed in 100 μl reaction system consisting of a final concentration of 0.5 mM *p*-NP esters substrate and the buffer (pH 8.0) of 80 mM H_3_BO_3_, 80 mM H_3_PO_4_, 300 mM NaCl, 0.3% Triton X-100 and 20% glycerol. The reaction mixture with increasing concentrations gradient (1, 2, 3, and 4 μg) of purified protein was incubated at 37°C for 40 min and the release of *p*-NP was determined by measuring spectrophotometrically at 405 nm. One unit of enzyme activity was defined as the amount of enzyme releasing 1 μmol of *p*-NP per min at 37°C under standard reaction conditions.

Substrate specificity was determined by using *p*-NP esters with different aliphatic side chains (C_2_-C_18_), as mentioned above. The optimum temperature of the enzyme reaction was determined in the same substrate solution at various temperatures ranging from 35°C to 60°C. The optimal pH for lipase activity was determined by assaying the hydrolytic activity at pH ranging from 6.0 to 9.0, using an established spectrophotometric method [16]. The effects of nonionic detergent Tween 20 and Tween 80, anionic detergent SDS and serine proteinase inhibitor phenylmethylsulfonyl fluoride (PMSF, Sigma-Aldrich) on lipase activities were examined by adding increasing concentrations gradient of each into the reaction system. All reactions were conducted in triplicate.

### Molecular modeling

The 3D model of LipL was generated using SWISS-MODEL (http://swissmodel.expasy.org/). Briefly, a new modeling project was started, and the target sequence file in the FASTA format was uploaded to search for templates. The top-ranked structure based on similarity with the target sequence was selected as the template structure. A 3D model of LipL was constructed with template by the Modeler module. The structural figures were prepared using the program PyMOL (http://www.pymol.org/).

### Subcellular fractionation of mycobacteria and immunoblotting

The subcellular fractionation of *M*. *smegmatis* was performed as described by Carolina Vizcaino *et al*. with minor modifications [19]. Briefly, *M*. *smegmatis* cells were harvested by centrifugation at 8,000 g for 30 min and washed twice in ice-cold phosphate buffered saline (PBS). Then, 10 g (wet weight) of the pellets was suspended in 20 ml of ice-cold PBS containing DNase, RNase, and proteinase inhibitor cocktail (Sigma-Aldrich). The suspension was passed through a high-pressure cell disruptor, and the resulting lysates were centrifuged at 3,000 g to remove any unbroken cells. The lysate supernatant was centrifuged at 27,000 g at 4°C for 1 hour, and the resulting supernatant was transferred into conical tubes (50 ml, Nest). The pellet was resuspended in lysis buffer containing 1 mM PMSF and 2 mM lysozyme (Sigma-Aldrich), and centrifuged at 27,000 g at 4°C for 1 hour to obtain the pellet, which mostly comprised of the cell wall fraction (CW). The supernatants obtained from the above-described two centrifugations were pooled together and centrifuged at 100,000 g and 4°C for 4 hours. The cytoplasmic (Cy) fraction was the supernatant obtained from this centrifugation. A final centrifugation at 100,000 g and 4°C for 4 hours was performed to remove the any cell membrane contaminant. Both pellets were pooled and considered cell membrane (CM) fraction. All of the fractions were resuspended in 10 mM NH_4_HCO_3_ buffer.

The protein concentrations of each fraction were determined using the BCA protein assay kit (Pierce, USA). Equal amounts of each fraction (20 μg) were separated by 12% SDS-PAGE and transferred onto a nitrocellulose membrane (Pall Corporation, NY, USA). The membranes were then blocked with 5% skimmed milk in PBS + 0.05% Tween 20 (PBST) at 37°C for 2 hours. The membranes were exposed to mouse anti-His-tag mAb at 1/2,000 dilution or rabbit anti-KatG antiserum at 1/1,000 dilution and incubated for 1 hour at 37°C. After washing three times for 10 min each, the membranes were incubated with Dylight 680-labeled goat anti-mouse IgG antibody or Dylight 800-labeled goat anti-rabbit IgG antibody (KPL, Gaithersburg, MD, USA) at 1/10,000 dilution, respectively. Antiserum against *M*. *tuberculosis* KatG was used as a quality control to ensure that there was no cytoplasmic contamination in the other fractions. The bands were detected using the Odyssey infrared imaging system (LI-COR Biosciences) in both the 700 nm and 800 nm channels.

### Study population and serological characterization

Sera samples were obtained from 51 TB patients who were admitted to the Heilongjiang Provincial Hospital for Prevention and Treatment of Tuberculosis, Harbin, China, and 45 clinically healthy donors. The diagnoses of TB patients were confirmed by examination of sputum-positive smears for acid-fast bacillus. The patients with pulmonary TB were categorized into two groups as follows. Group 1 (n = 29) comprised patients who had been diagnosed with TB for the first time and had no history of TB treatment. Group 2 (n = 22) comprised relapsed TB patients who were previously treated for TB and disease relapsed after the initial completion treatment. Clinically healthy donors were those who showed no symptoms of TB and had been vaccinated with *M*. *bovis* BCG during infancy. All of the sera samples were negative for immunodeficiency virus.

Enzyme-linked immunosorbent assays (ELISAs) were performed in 96-well plates (Nest) and coated with LipL_Ms_ purified from recombinant *M*. *smegmatis* strains and LipL_E_ purified from recombinant *E*. *coli*. The plates were incubated with the protein (5 μg/ml) diluted in coating buffer (50 mM NaHCO_3_/Na_2_CO_3_, pH 9.6) overnight at 4°C. The plates were washed three times with PBST. After blocking with 5% skimmed milk in PBST, the plates were washed and incubated with human sera (1:200 dilution in PBST) at 37°C for 1 hour. After washing three times, the plates were further incubated with goat anti-human IgG antibody (Rockland, Gilbertsville, PA, USA) conjugated to HRP at 1:20,000 dilution. After washing, the substrate 3, 3’, 5, 5’-tetramethylbenzidine (TMB) (Sigma-Aldrich) was used to detect the HRP activity, and the reactions were terminated using 0.25% hydrofluoric acid (Amresco). The absorbance values were measured at 630 nm using a BioTek ELx808 Absorbance Microplate Reader (BioTek, Winooski, VT, USA).

### Immunization of mice, flow cytometry analysis for spleen lymphocytes and statistical analysis

The BALB/c mice used in this study were housed in 4 groups and given one week to acclimate to the housing facility. Mice were maintained in cages fitted with micro-isolators connected to air circulator. The cages had bedding, feed and water. The environmental temperature, humidity, and light were in the standard conditions conducive to mice activity. During housing, mice were monitored every day for health status and no adverse events were observed. Breeder was cleaned in the morning every day. A completed ARRIVE guidelines checklist is included in S1 Checklist.

In this study, 5–6 weeks old female BALB/c mice (Vital River, Beijing) maintained under specific pathogen-free conditions were used. Mice were randomly divided into 2 groups. Groups of mice (n = 6 per group) were immunized with the LipL_Ms_ recombinant protein and PBS (mock-immunized). LipL_Ms_ was purified from recombinant *M*. *smegmatis* strains. The mice were immunized at the age of 6–7 weeks by the subcutaneous injection of 100 μg of recombinant protein in a 200 μl volume formulated in protein plus incomplete Freund’s adjuvant (IFA) or PBS plus IFA. The mice were immunized two times at an interval of 14 days. The spleens of the mice were harvested 14 days after the second immunization.

The mice were allowed to rest for two weeks after the secondary immunization. Mice were euthanized by cervical dislocation in the laboratory and the spleens were then removed aseptically. Single-cell spleen suspensions were prepared aseptically, and spleen lymphocytes were obtained using a mice spleen lymphocyte separation reagent kit (TBD). The spleen lymphocytes were washed three times in PBS and resuspended in RPMI-1640 supplemented with 20% fetal bovine serum (FBS, Gibco) and 1% penicillin-streptomycin. The cells were then counted, and adjusted to a final concentration of 1 × 10^6^ cells/ml and grown in 24-well plates. The cells were stimulated with the corresponding purified protein at a concentration of 10 μg/ml and incubated at 37°C for 12 hours. The cells were then washed twice and incubated with specific surface antibodies, including anti-CD3-APC, anti-CD4-FITC, and anti-CD8-PE (Invitrogen, USA), for 1 hour at room temperature. The cells were washed twice and then processed for flow cytometry analysis (FACSAria flow cytometer, BD Biosciences), and the data were analyzed using FlowJo software (http://www.flowjo.com/).

Each experiment was independently repeated three times. Two-tailed Student’s *t* test was used for the analysis of statistical significance (*P* value) in this study, and Prism software (version 5.0; GraphPad, San Diego, CA, USA) was used for these analyses. A *P* value of less than 0.05 was considered significant (* *P* < 0.05; ** *P* < 0.01; *** *P* < 0.001; ns, not significant).

## Results

### Cloning, expression, and purification of LipL

**Fig 1:**
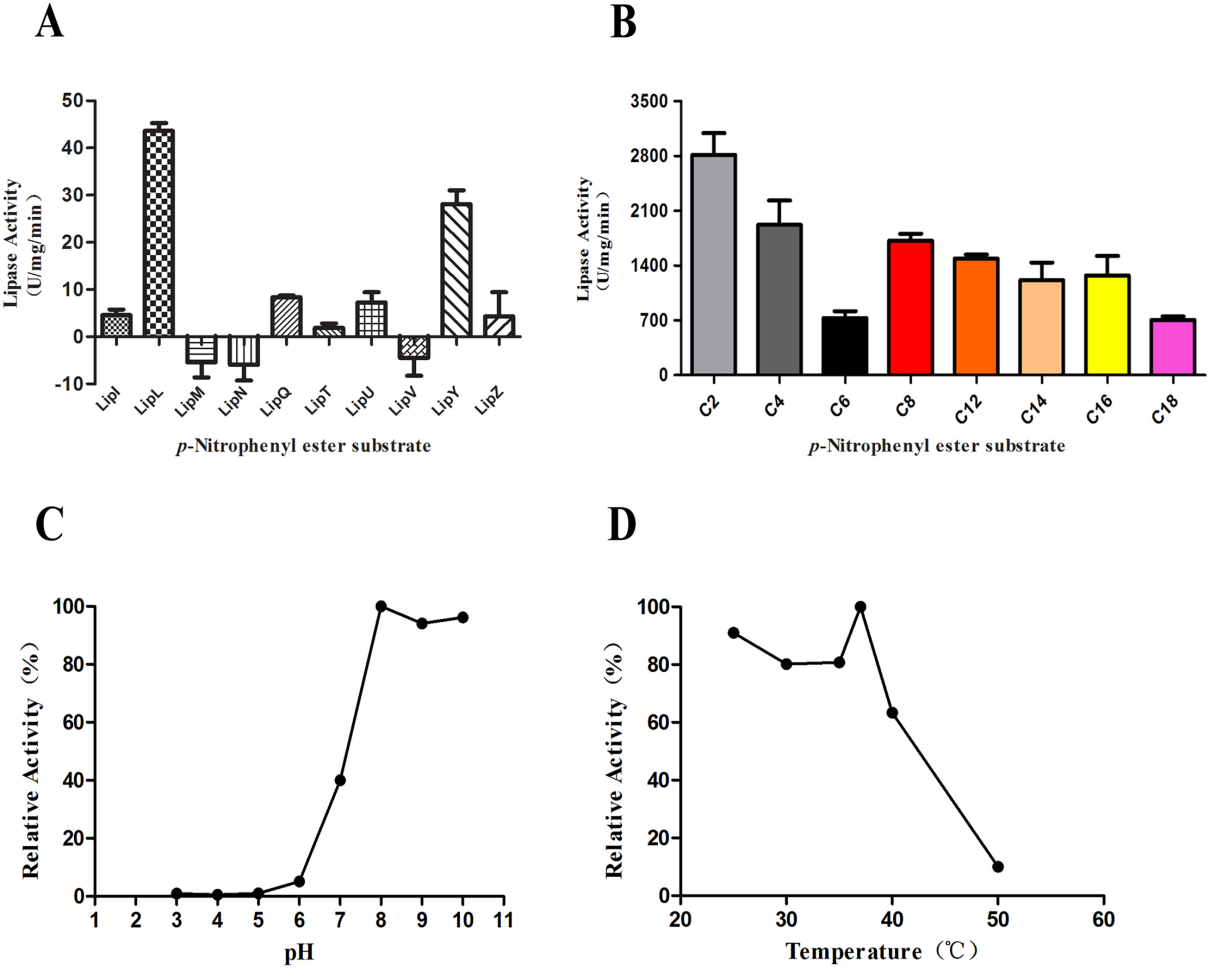
Analysis of lipase activity. (A) The total lipase activities were assayed using cell lysate supernatants from the 10 recombinant *M*. *smegmatis* strains. The *p*-NP palmitate (C_16_) was used as the substrate for the lipase activities analysis. The values represent the means ± standard deviations (SD) of three independent experiments. (B) Lipase activity of LipL towards *p*-NP esters with various chain lengths (C2, acetate; C4, butyrate; C6, caproate; C8, caprylate; C12, laurate; C14, myristate; C16, palmitate; and C18, stearate). The values represent the means ± SD of three independent experiments. (C) The effect of pH on lipase activity of LipL. (D) The effect of temperature on lipase activity of LipL.

Ten hypothetical esterases/lipases belonging to the Lip family of *M*. *tuberculosis* were overexpressed in *M*. *smegmatis*, and the total lipase activities were assayed using the cell lysate supernatants of recombinant *M*. *smegmatis* strains. The highest lipase activity was observed with the strain overexpressing the *lipL* gene (Fig 1A). Therefore, the *lipL* gene was selected for further investigation. The *M*. *tuberculosis lipL* gene encodes a protein comprising 429 amino acids with a molecular mass of 48.5 kDa and a calculated pI value of 8.5. We first cloned the *lipL* gene into the pET-28b vector, and the resulting recombinant protein was expressed in insoluble form. Although we experimented with different expression conditions, including different host strains, temperatures, media, and IPTG concentrations for induction, the recombinant protein was always expressed as inclusion bodies. We then attempted to obtain a renaturation product with biochemistry activity from the inclusion bodies using several renaturation conditions. However, active proteins were not obtained. Thus, we constructed the acetamide-inducible vector pAIN for *M*. *smegmatis*, in which mycobacterial proteins could be expressed in soluble form. We cloned the *lipL* gene into the pAIN vector to obtain the recombinant plasmid plipL. The LipL protein was expressed as a His-tagged fusion protein in *M*. *smegmatis* in both soluble and insoluble forms. The soluble component was purified by affinity chromatography on a Ni-NTA column, followed by an anion-exchange chromatography RESOURCE^TM^ Q column, and finally by Superdex 75. The samples were analyzed by SDS-PAGE, and the results showed that the purified protein had an expected molecular mass of approximately 48.5 kDa (S1A Fig).

To confirm that the purified proteins were LipL, Western blot analysis and high-performance liquid chromatography mass spectrometry (HPLC-MS) were performed. The Western blot analysis results showed that the purified product band that reacted with anti-His-tag antibodies was approximately 50 kDa, in agreement with the expected molecular weight of LipL (S1B Fig). The purified LipL protein was further validated by HPLC-MS analysis. The result obtained from searching NCBI databases using the HPLC-MC data matched LipL (**GenBank accession no. NP216013**) from *M*. *tuberculosis* H37Rv with a score of 292. The results of the Western blot and HPLC-MS assays confirmed that the purified protein was in fact LipL.

### Substrate specificity of LipL

Lipases, which hydrolyze long-chain esters, are considered much more crucial for the pathogenicity of *M*. *tuberculosis* than esterases, which can only hydrolyze short-chain esters. To investigate the substrate specificity of the purified LipL protein, we applied a wide range of esters, particularly the long-chain esters of *p*-NP (C_12_ to C_18_), as substrates in the enzymatic activity detection experiment. The substrates included *p*-NP acetate (C_2_), *p*-NP butyrate (C_4_), *p*-NP caproate (C_6_), *p*-NP caprylate (C_8_), *p*-NP laurate (C_12_), *p*-NP myristate (C_14_), *p*-NP palmitate (C_16_), and *p*-NP stearate (C_18_). The results showed that LipL displayed rather high activity against both long-chain and short-chain *p*-NP esters (Fig 1B). Therefore, the relatively high activity displayed by LipL with long-chain esters (C_12_ to C_18_) suggested that it could be classified as a lipase.

### Effect of pH and temperature on lipase activity of LipL

The lipase activities of LipL were analyzed within the pH range from 3.0 to 10.0 and the temperature range from 25°C to 50°C using *p*-NP laurate (C_12_) as the substrate. The optimal activity was detected at pH 8.0 and 37°C (100% relative activity). LipL possessed activities over a range of pH values from 7.0 to 10.0, but had reduced activity in acidic conditions with pH values from 3.0 to 7.0 (Fig 1C). LipL also displayed activities over a broad range of mild temperatures from 25°C to 50°C (Fig 1D). LipL retained 91%, 63%, and 10% relative activity at 25°C, 40°C, and 50°C, respectively. This finding indicated that lower temperatures favored LipL lipase activities while temperatures greater than 40°C severely damaged LipL lipase activities.

### Effects of detergents on LipL activity

**Fig 2:**
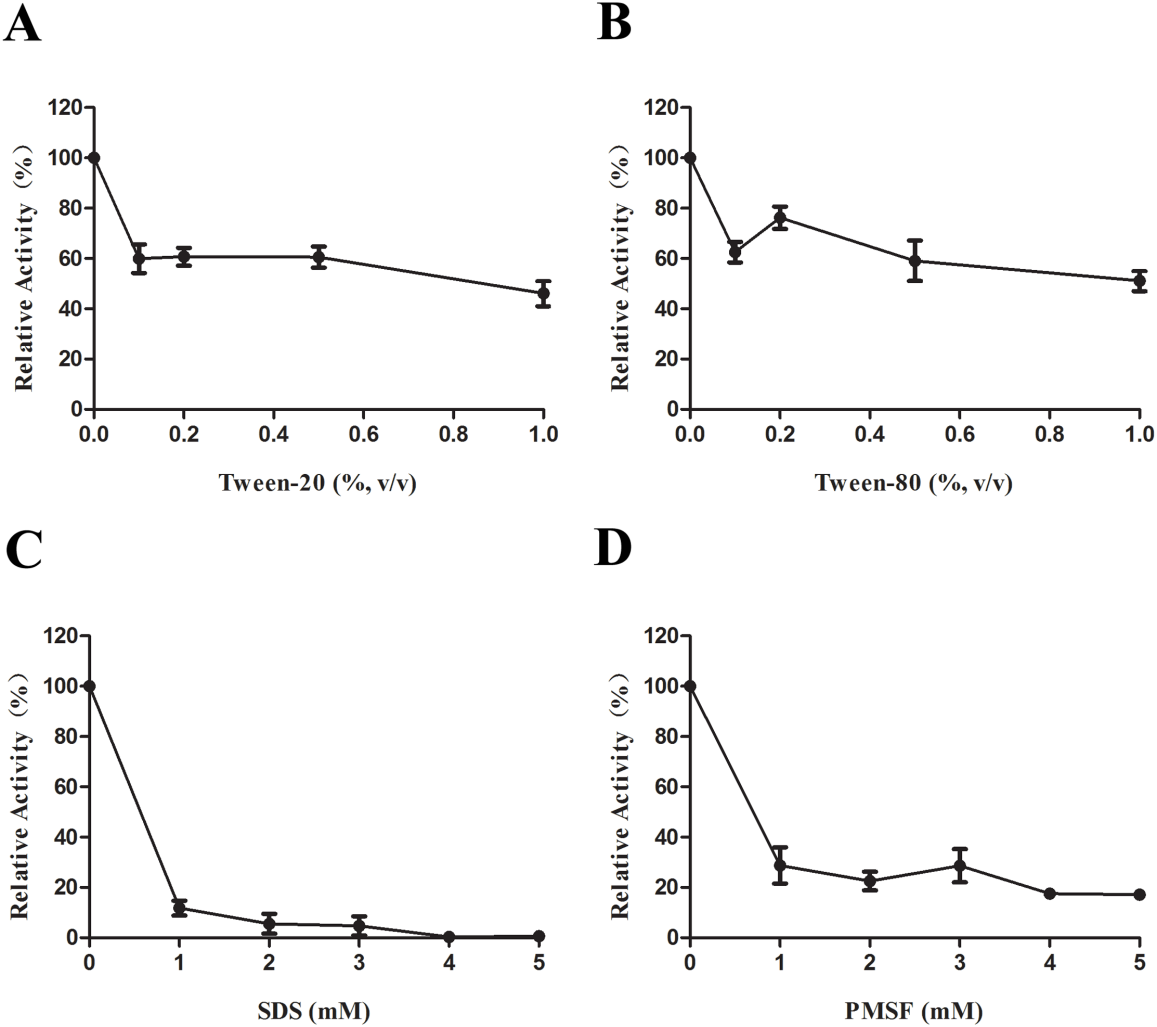
The effect of detergents on lipase activity of LipL. (A) Addition of Tween 20 at concentration ranging from 0%–1% in the LipL lipase activity assays. (B) Addition of Tween 80 at concentration ranging from 0%–1%. (C) Addition of SDS at concentration ranging from 0 mM–5 mM. (D) Addition of PMSF at concentration ranging from 0 mM–5 mM. Data represent the means ± SD of three independent experiments.

We next assessed the effect of various detergents on LipL lipase activity. The addition of the nonionic detergent Tween 20 or Tween 80 resulted in reductions in the relative lipase activity by approximately 40% (Fig 2A and 2B). The most prominent reductions in the relative lipase activities of approximately 85% and 75% were observed upon the addition of the anionic detergent SDS and the serine proteinase inhibitor PMSF, respectively (Fig 2C and 2D).

### Site-directed mutagenesis

LipL contains the G-x-S-x-G motif, which represents the characteristic feature of the α/β hydrolase-fold family. In addition to the most common motif G-x-S-x-G, the GGG and S-x-x-K motifs, which were recently discovered to have a direct linkage to enzymatic activity, were also present in the LipL amino acid sequence. To identify the residues in the LipL primary structure that are necessary for catalytic activity, site-directed mutagenesis of the important residues within the above-mentioned three motifs was performed.

**Fig 3:**
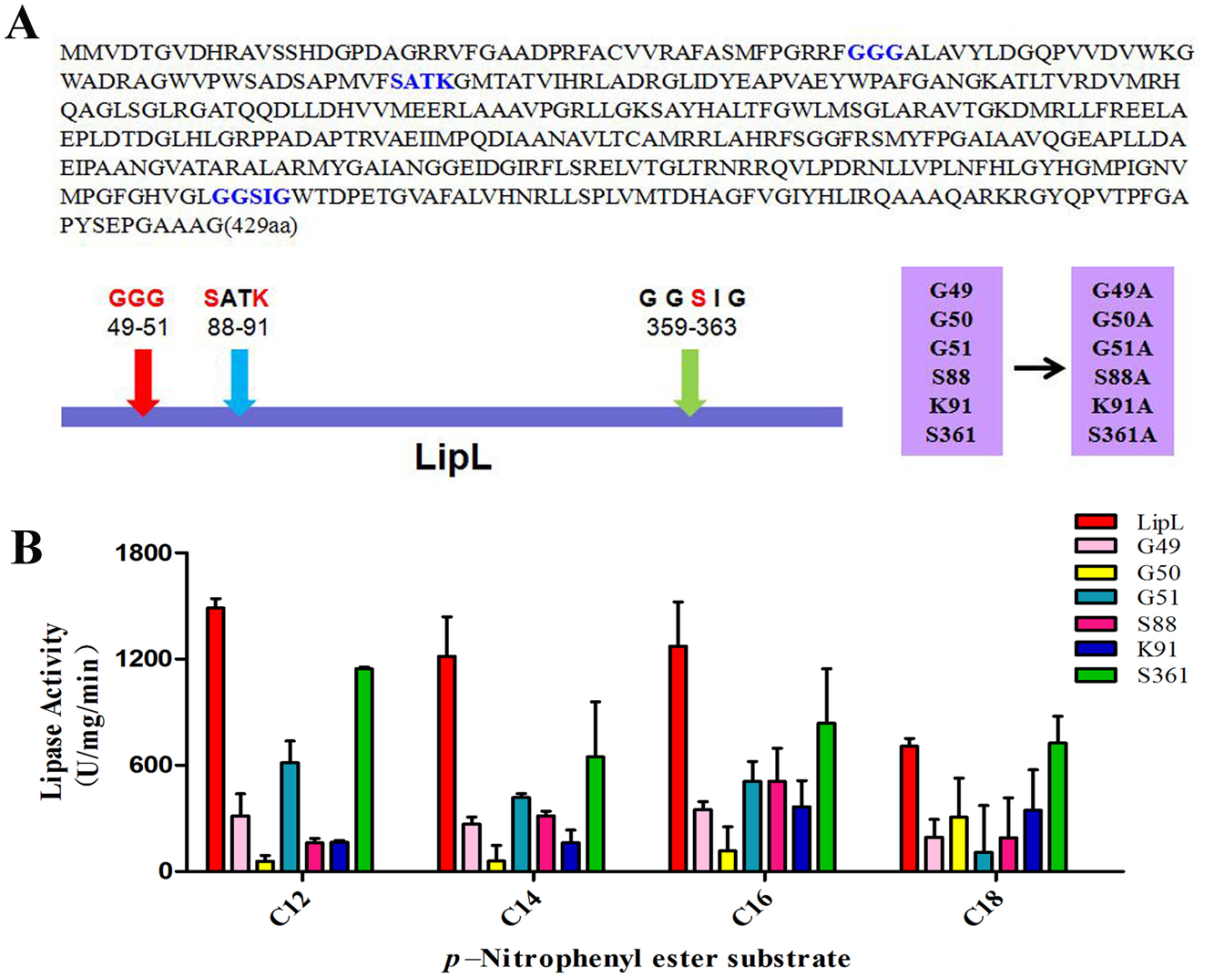
The analysis of the lipase activities of the mutants. The six residue substitutions of site-directed mutagenesis were G49A, G50A, G51A, S88A, K91A, and S361A. (A) A schematic of the LipL protein sequence with predicted catalytic motif and the mutations. (B) The experiment was performed in the standard lipase activity assays. The values represent the means ± SD of three independent experiments.

Six residues of the three motifs were replaced to determine which residues constituted the catalytic center of LipL essential for lipase activity. The proposed mutations, including G49A, G50A, G51A, S88A, K91A, and S361A (Fig 3A), were generated by PCR using the template plasmid pAIN-lipL and the mutagenic oligonucleotide pairs. The alanine residue was chosen as a substitute residue because it lacked a bulky side chain and therefore would likely not have any steric and electrostatic effects. Moreover, it would not destroy the main-chain conformation even in the presence of other residues. The analysis of the activities of the mutants compared with that of the wild-type LipL showed that each of the G50A, S88A, and K91A substitutions resulted in an approximately 90% loss of activity, whereas the G49A and G51A substitutions resulted in losses in activities of 70% and 60%, respectively (Fig 3B). The S361A substitution resulted in an approximately 20% loss of lipase activity. In general, the G50 residue in the GGG motif and the S88 and K91 residues in the S-x-x-K motif showed a significant impact on the lipase activity. Therefore, we considered the GGG and S-x-x-K motifs as the catalytically central part of LipL and that, the G50, S88, and K91 residues in these motifs were essential for the lipase activity.

### 3D Model of LipL

Because the GGG and S-x-x-K motifs were identified as crucial LipL for lipase activity and specifically residues G50, S88, and K91 were determined to be catalytically critical, to further obtain insights into the possible location of the catalytic residues within the overall topological organization of LipL, a 3D model was built using the SWISS MODEL server with EstA carboxylesterase, which possesses the highest sequence homologies with LipL, as the template (PDB code: 3ZYT).

**Fig 4:**
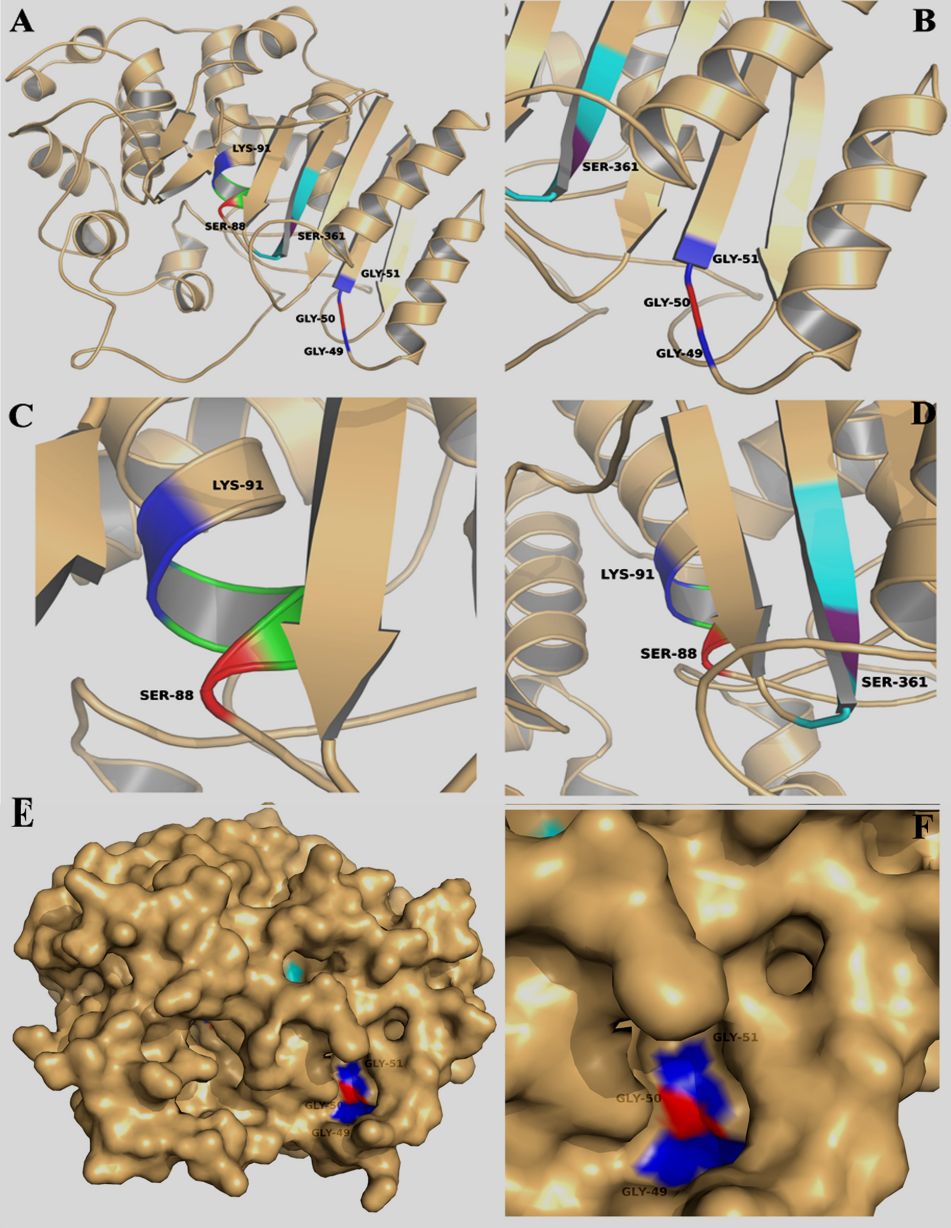
Homology model of LipL (template: EstA carboxylesterase, PDB code: 3ZYT). (A) Ribbon view of LipL model which consisted of several α helices and β sheets. The predicted active residues are shown in different colors. (B) Enlarged view of GGG motif (residues G49 and G51 are shown in blue; residue G50 is shown in red). (C) The close-up view of the S-x-x-K motif (residue S88 is shown in red; residue K91 is shown in blue). (D) Enlarged view of the G-x-S-x-G motif (residue S361 is shown in purple). (E) The partially transparent surface representation of LipL. The GGG motif is highlighted shown in blue and red colors. It is noteworthy that the GGG motif is formed a catalytic pocket surface structure. (F) The close-up view of the surface representation of the GGG motif.

As expected, the model (Fig 4A) showed that LipL consisted of several α helices and β sheets, and this result was in agreement with the α/β hydrolase-fold family. The six predicted critical residues are clearly shown in the ribbon diagram of LipL. The secondary structures of the predicted motifs that form the catalytic center of lipase activity are displayed in the next three ribbon diagrams. The GGG motif is located in the junction of an α helix and a β sheet, and the G50 residue is located in the center of this motif (Fig 4B). The partially transparent surface representation of the GGG motif enabled us to better visualize the location of this motif (Fig 4F). A substrate binding pocket was observed in the surface of the GGG motif (blue and red colors). These features of the GGG motif are consistent with our biochemical results. The S-x-x-K motif is located in a helix structure (Fig 4C); the S88 residue is shown in red color, and the K91 residue is depicted in blue. Fig 4D shows the conserved G-x-S-x-G motif, which is located in a β sheet, and the S361 residue is shown in purple. In fact, the whole protein with a partially transparent surface was constructed (Fig 4E). It is noteworthy that the GGG motif does in fact form a catalytic pocket surface structure. However, the surface structures of the S-x-x-K and G-x-S-x-G motifs are hidden from sight because they are inside the protein. The close-up view of the surface structure of the GGG motif is highlighted in Fig 4F.

### Subcellular location of LipL in mycobacteria

**Fig 5:**
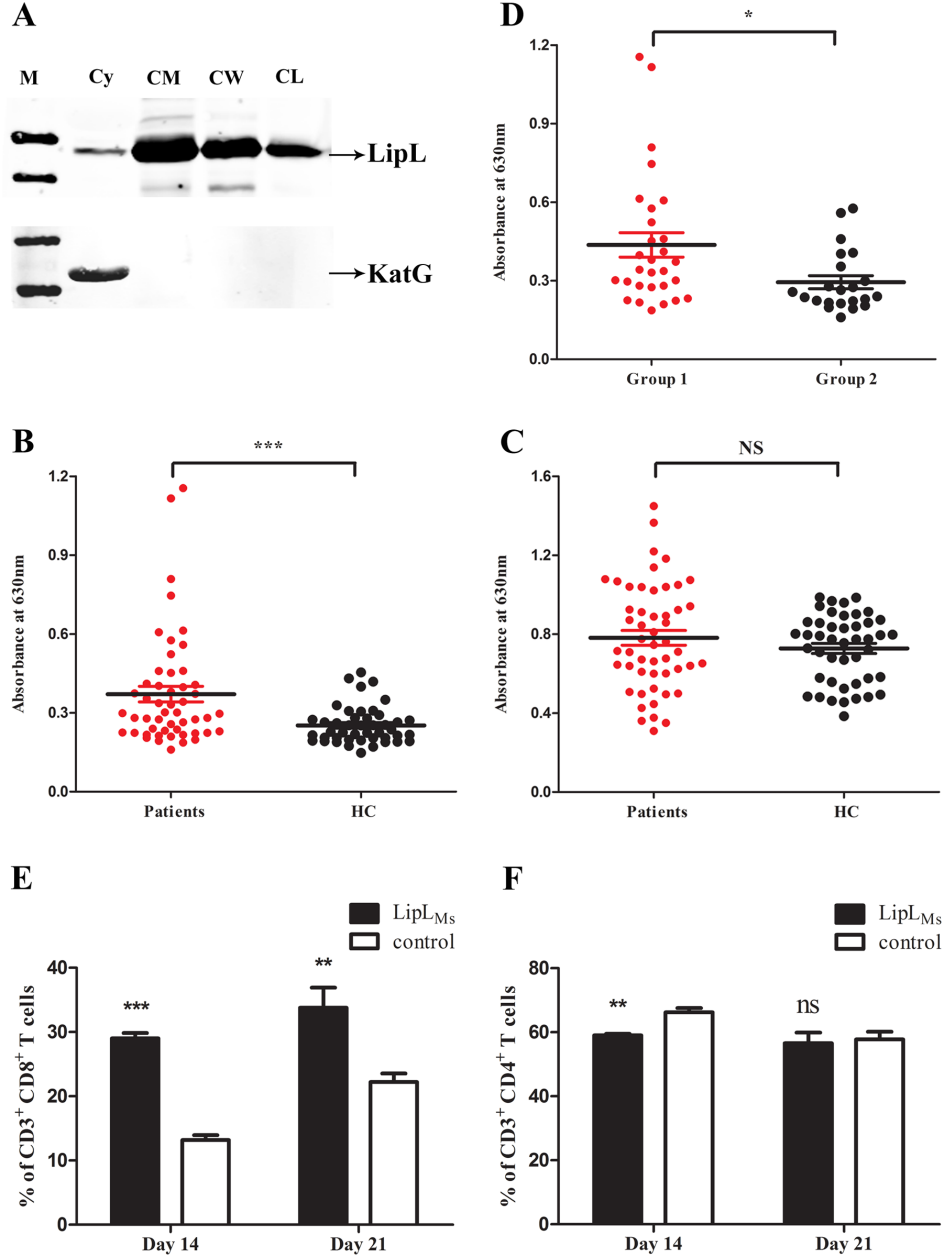
Subcellular localization, the humoral and cell-mediated immune responses of LipL. (A) Subcellular localization of LipL in *M*. *smegmatis*. Bacteria were lysed and fractionated to separate the cytoplasm (Cy) from the cell wall (CW). Equal amounts of protein (20 μg) from each fraction were subjected to SDS-PAGE, transferred onto a nitrocellulose membrane, and probed with either monoclonal anti-His antibodies (top) or rabbit anti-KatG antiserum (bottom). (B and C) The humoral responses induced by the recombinant LipL_Ms_ (B) and LipL_E_ (C) proteins in TB patients compared with healthy donors. The plates were coated with purified LipL_Ms_ or LipL_E_. ELISA reactions of LipL_Ms_ and LipL_E_ to the sera of either TB patients or healthy controls (HC) were assayed (n = 51 for patients; n = 45 for healthy controls). (D) ELISA reaction of LipL_Ms_ to the sera of either TB group 1 or group 2 patients were assayed (n = 29 for group 1; n = 22 for group 2). Student’s *t* test was used for analysis of statistical significance (*P* value). Only *P* values of < 0.05 were considered significant (* *P* < 0.05; ** *P* < 0.01; *** *P* < 0.001; ns, not significant). (E) Graphical representation of spleen CD3^+^ CD8^+^ T cell percentages from LipL_Ms_ and PBS immunized mice. (F) Graphical representation of spleen CD3^+^ CD4^+^ T cell percentages from LipL_Ms_ and PBS immunized mice. The data represent the means ± standard errors of the means (SEM) of three independent experiments, and the statistically significant differences were revealed using unpaired Student’s *t* test (* *P* < 0.05; ** *P* < 0.01; *** *P* < 0.001; ns, not significant).

To investigate the subcellular location of LipL in mycobacteria, subcellular fractions prepared from *M*. *smegmatis* overexpressing LipL were probed with anti-His monoclonal antibody to detect the presence of this protein. The possible contamination of the cell wall components by cytoplasmic components was excluded by probing the fractions with antiserum against KatG of *M*. *tuberculosis*. The presence of KatG in the cytoplasmic fraction and its absence in the cell wall fraction validated the purity of the cell wall preparations. The Western blot results showed that the protein bands that exhibited strong intensity were recognized in the cell wall and the cell membrane fractions, but was also detected in cytoplasmic fraction albeit at a weaker intensity (Fig 5A and S2 Fig). This result suggested that LipL was present in the cell wall and the cell membrane, and to a lesser extent in the cytoplasm.

### Immunogenicity of LipL

The results of the subcellular localization experiment suggested that LipL was mainly located in the cell membrane and cell wall. We therefore hypothesized that LipL may be accessible to the host immune system. To investigate whether the host humoral immune system would react to LipL, an indirect ELISA was performed to evaluate the reaction of the sera of TB patients using the recombinant protein LipL_Ms_, which was expressed and purified from *M*. *smegmatis*. Because we primarily obtained purified LipL_E_ from the *E*. *coli* expression system, which showed no biochemical activity, the difference in the immuno-reactivities of LipL_Ms_ and LipL_E_ was also determined through ELISA assays.

The data revealed that the sera of all infected patients mounted significantly higher antibody responses against LipL_Ms_ compared with those obtained from the healthy controls (*p* = 0.0006; Fig 5B). However, in contrast to LipL_Ms_, the use of LipL_E_ as the detection antigen resulted in no significant differences in the sera reactions between TB patients and healthy controls (*p* > 0.05; Fig 5C). The overall reactions of all of the sera samples to LipL_E_ were stronger than those to LipL_Ms_ (Fig 5C). The results clearly indicated that a LipL-specific humoral immune response was induced in TB patients. The results also inferred that LipL_E_ could barely react with the LipL-specific antibody, indicating that it lost its immuno-reactivity as well as its biochemical activity. Because the patient samples included sera from two groups of newly infected patients and relapsed patients, we also assayed the difference in the B cell responses elicited by these two groups against LipL_Ms_. The results demonstrated that the sera from the newly infected group responded better than those from the relapsed group (*p* = 0.0173; Fig 5D).

### Relative proportion of spleen CD4^+^ and CD8^+^ T cell subsets

The immunogenicity experiments suggested that LipL protein provoked strong humoral responses during TB infection. We then aimed to determine whether LipL protein could elicit a cell-mediated immune response *in vivo*. BALB/c mice were subjected to either PBS (mock) or LipL_Ms_ recombinant protein immunization. There were no adverse effects after either mock or LipL protein immunization. Splenocytes from mice immunized with LipL_Ms_ were isolated 2–3 weeks after the last immunization and stimulated with LipL_Ms_ protein in a 24-well plate for 12 hours. The splenocytes were harvested, stained with fluorescein labeled antibodies, and subjected to flow cytometry to determine the percentages of CD3^+^ CD8^+^ and CD3^+^ CD4^+^ T cells.

Two weeks after protein immunization, the number of CD3^+^ CD8^+^ T cells in the LipL_Ms_-immunized mice increased, and the percentage was significantly higher than that of the control group (*p* < 0.0001; Fig 5E and S3A Fig), whereas the number of CD3^+^ CD4 T cells in the LipL_Ms_ group was significantly decreased compared with the control group (*p* = 0.006; Fig 5F and S3B Fig). Three weeks after immunization, the percentage of CD3^+^ CD8^+^ T cells in the LipL_Ms_-immunized group was also significantly higher than that in the control group (*p* = 0.0066; Fig 5E and S3A Fig), while the difference in the number of CD3^+^ CD4^+^ T cells in the LipL_Ms_ group compared with the control group was not significant (*p* > 0.05; Fig 5F and S3B Fig).

Our data demonstrated that the LipL protein can effectively activate CD8^+^ T cell-mediated response, which was stronger than the CD4^+^ T cell response. These results suggested that LipL could elicit significantly strong cell immune responses and particularly a cytotoxic effect in mice.

## Discussion

Although it is well known that esterases/lipases are crucial for the pathogenicity of mycobacteria [18], the esterases/lipases from mycobacteria have not been sufficiently characterized. This could, at least partly, be due to the difficulty in obtaining soluble and biochemically active esterase/lipase products in the *E*. *coli* expression system [6, 16, 20]. Esterases/lipases usually form inclusion bodies without biochemical activity in the *E*. *coli* expression system, and their enzymatic activities cannot be observed unless they are successfully renatured [15, 17, 21–23]. Primarily, we attempted to express the members of the Lip family of *M*. *tuberculosis* in the *E*. *coli* system. All of the recombinant proteins formed inclusion bodies and failed to renaturate. To obtain natural and biochemically active products, a robust and acetamide-inducible expression vector for the host bacterium *M*. *smegmatis*, denoted pAI, was constructed as described previously [24, 25].

We overexpressed 10 hypothetical esterases/lipases belonging to the Lip family of *M*. *tuberculosis* in the *M*. *smegmatis* expression system. The total lipase activities were assayed using the cell lysate supernatants from the 10 recombinant *M*. *smegmatis* strains using a previously described method [16]. Interestingly, we found that the highest lipase activity was observed with the strain overexpressing the *lipL* gene (Fig 1A), which was markedly higher than that obtained for the positive control LipY, the most intensively studied lipase in *M*. *tuberculosis* [15, 17, 26–31]. Therefore, we focused on the LipL protein in the subsequent studies. The substrate specificity of LipL was investigated through lipase activity assays using purified LipL_Ms_ from *M*. *smegmatis*. Meanwhile, experiments with LipY were performed as the positive control (S4 Fig). The results showed that LipL could not only hydrolyze short-chain esterases but also had a rather high activity against long-chain lipids (Fig 1B). The optimal temperature for enzyme activity was found to be 37°C, and the optimal pH was identified as 8.0. Both of these characteristics reflect the adaptation of mycobacteria to the host environment.

Of these 10 recombinant proteins, six had the G-x-S-x-G consensus sequence, which is considered a characteristic feature of the α/β hydrolase-fold family [21]. In addition, five of these proteins had the S-x-x-K motif, which is conserved in the carboxylesterase VIII family [32], and four had the GGG motif, which had been reported in certain esterases [33]. Among the 10 proteins, while some had one or two of the three motifs, LipL had all three motifs. To determine which motif was responsible for the lipase activity of LipL, a site-directed mutagenesis experiment was performed. The lipase activity detection of the mutants showed that G50A, S88A, and K91A substitutions resulted in almost 90% loss of lipase activity compared with the wild-type protein (Fig 3B). This result indicated that the lipase activity was related to the S-x-x-K and GGG motifs. In addition, the key amino acid residues of the active center of the lipase were determined through the site-directed mutagenesis. Through homologous modeling, we found that the GGG motif formed the pocket structure on the three dimensional surface (Fig 4E and 4F). A previous study revealed that the GGG motif was considered an oxyanion hole motif, which is consistent with the findings of our modeling study [33]. Furthermore, the ribbon diagram showed that LipL was composed of multiple α helices and β sheets structure (Fig 4A), further suggesting that LipL belongs to the α/β hydrolase-fold family. We revealed the overall topological organization of LipL through homology modeling, and the predicted active center was consistent with the experimental results of the site-directed mutagenesis experiment. This finding provides information that has important reference value for further detailed analysis of the structure and function of LipL.

Although the LipL protein had no predicted signal peptides according to the SignalP 3.0 tool, which indicated that LipL is not secreted *via* the classical pathway, this finding cannot exclude the possibility of its secretion through non-classical mechanisms. Regarding non-classical secretion mechanisms, important secretory proteins, such as ESAT-6 and CFP-10, also contain no signal sequence in their N-termini. However, LipL was predicted to be an exported protein by the Gpos-PLoc (http://www.csbio.sjtu.edu.cn/bioinf/Gpos-multi/), Phobius (http://www.ebi.ac.uk/Tools/pfa/phobius/), and PSORTb 3.0.2 (http://www.psort.org/psortb/index.html) tools [19, 34]. The result from the Western blot analysis of the mycobacterial subcellular fractions showed that LipL was located on the cell wall and cell membrane, suggesting the likelihood of the secretion of LipL.

Proteins expressed on the bacterial surface are accessible to the host’s humoral or cellular immune system and are therefore important humoral or/and cellular immunogens [35–37]. Given its subcellular location in mycobacteria, we thus hypothesized that LipL could induce host humoral or cell-mediated immune responses. The strong humoral response against LipL elicited in TB patients indicated that LipL was expressed during the infection period in TB patients and LipL has good immunogenicity. According to the antigen-antibody reaction results, LipL_E_ lost its immuno-reactivity compared with LipL_Ms_. The reason might be due to the LipL_E_ could not correctly fold *via* resolution from an inclusion body, which suggested that the immunogenicity of LipL was mostly derived from spatial epitopes and that a probable linear conformation of LipL_E_ could not appropriately react with LipL-specific antibodies. This finding also suggested that the use of the *M*. *smegmatis* expression system was a good strategy for the expression and purification of mycobacterial antigens for the development of both immuno-diagnostic methods and vaccine components. Similarly, the *M*. *smegmatis* expression system will be more practical for obtaining soluble and natural mycobacterial proteins during the functional characterization of a gene from *M*. *tuberculosis* [25, 38].

The sera samples from TB patients used in our study represented a heterogeneous population, including newly infected cases and relapsed cases. The immune response profiles of LipL in the two groups were assessed. A previous report emphasized a lack of sufficient immune responses against many *M*. *tuberculosis* antigens in newly infected cases [39]. Surprisingly, in our study, the newly infected patients (group 1) responded better against LipL than the relapsed patients (group 2). This difference may be attributed to the difference in immunogenicity among *M*. *tuberculosis* antigens. Alternatively, it is also likely that the group 2 patients could not induce sufficient immune responses against TB, resulting in relapse.

CD4^+^ T cells and CD8^+^ T cells are crucial components of acquired immune responses and are important for protective immunity against *M*. *tuberculosis*. CD4^+^ T cells produce cytokines to activate macrophages, while CD8^+^ T cells recognize antigenic peptides loaded in the cytosolic compartment and capable of effecting anti-microbial mechanisms, which are absent or less prominent in CD4^+^ T cells [40]. Although the role of CD8^+^ T cells in TB is less understood than that of CD4^+^ T cells, CD8^+^ T cells were recently hypothesized to markedly contribute to optimal immunity and protection against *M*. *tuberculosis* [41–43]. In our study, the analysis of the cellular immune response elicited by LipL in mice showed that the number of CD8^+^ T cells was significantly increased, whereas the number of CD4^+^ T cells was not changed. This finding suggested that the LipL protein could effectively activate the CD8^+^ T cell-mediated response, which may contribute to protective immunity against TB. Taken together, the results showed that the *M*. *tuberculosis* lipase LipL could induce both B and T cell immune response, thus LipL could potentially be used as a target for the development of clinical diagnostic reagents and as a vaccine candidate antigen alone or in combination with other immunogenic antigens for TB vaccine design. In addition, LipL was up-regulated in *M*. *tuberculosis* cultured under hypoxic conditions for 12 days [17]. We speculated that LipL provides a potential target for drug screening.

## Conclusions

The LipL protein was successfully expressed in and purified from *M*. *smegmatis*, and its characterizaiton demonstrated that it was a lipase with high enzymatic activity to hydrolyze long-chain lipids. The key amino acid residues of the active center of LipL were determined through site-directed mutagenesis and homologous modeling experiments. Given its potential surface location in mycobacteria, LipL was demonstrated to induce a strong humoral immune response and activate a CD8^+^ T cell-mediated response.

## Supporting Information

S1 Checklist Completed “The ARRIVE Guidelines Checklist” for reporting animal data in this manuscript. (PDF)

S1 Fig The purification and Western blot analysis of LipL_Ms_ from *M*. *smegmatis*. (A) Analysis of purified LipL_Ms_ protein by SDS-PAGE. Proteins were eluted with elution buffer at a gradient concentration of imidazole. Lanes: 1, eluate eluted with 100 mM imidazole; 2, eluate eluted with 200 mM imidazole; 3, eluate eluted with 500 mM imidazole; M, molecular mass markers. (B) LipL protein was confirmed by Western blot. The protein band reacted with His-tag antibodies. Lanes: 1–2, eluate eluted with 200 mM imidazole; 3–4: eluate eluted with 500 mM imidazole; M, molecular mass markers.
(TIF)

S2 Fig Subcellular localization of LipL in *M*. *smegmatis*. Bacteria were lysed and fractionated to separate the cytoplasm (Cy) from the cell wall (CW). Equal amounts of protein (20 μg) from each fraction were subjected to SDS-PAGE, transferred onto a nitrocellulose membrane, and probed with either monoclonal anti-His antibodies (top) or rabbit anti-KatG antiserum (bottom).
(TIF)

S3 Fig Cell-mediated immune response of LipL. Histograms showed the CD3^+^ CD8^+^ T cell subsets and the CD3^+^ CD4^+^ T cell subsets from LipL_Ms_ and PBS immunized mice. (A) The percentage of CD3^+^ CD8^+^ T cells in the LipL_Ms_ group was significantly higher than that of the control group. (B) The percentage of CD3^+^ CD4^+^ T cells in the LipL_Ms_ group was significantly decreased compared with the control group (day 21 not significant).
(TIF)

S4 Fig The lipase activity of LipY. Lipase activity of LipY towards *p*-NP esters with various chain lengths (C2, acetate; C4, butyrate; C6, caproate; C8, caprylate; C12, laurate; C14, myristate; C16, palmitate; and C18, stearate). The values represent the means ± SD of three independent experiments.
(TIF)
