# Supplementary figures and images for: Identification and Characterization of Lipase Activity and Immunogenicity of LipL from Mycobacterium tuberculosis
Source: PLoS One. 2015 Sep 23;10(9):e0138151. doi: 10.1371/journal.pone.0138151 (PMC4580317; doi:10.1371/journal.pone.0138151)

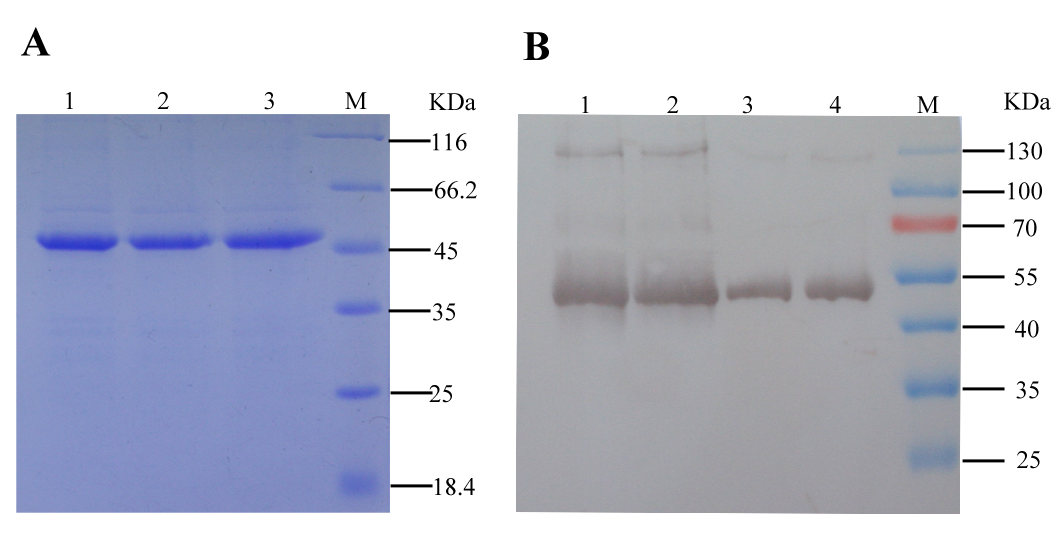

Supplement: S1 Fig — (A) Analysis of purified LipLMs protein by SDS-PAGE. Proteins were eluted with elution buffer at a gradient concentration of imidazole. Lanes: 1, eluate eluted with 100 mM imidazole; 2, eluate eluted with 200 mM imidazole; 3, eluate eluted with 500 mM imidazole; M, molecular mass markers. (B) LipL protein was confirmed by Western blot. The protein band reacted with His-tag antibodies. Lanes: 1–2, eluate eluted with 200 mM imidazole; 3–4: eluate eluted with 500 mM imidazole; M, molecular mass markers. (TIF) [file pone.0138151.s002.tif]

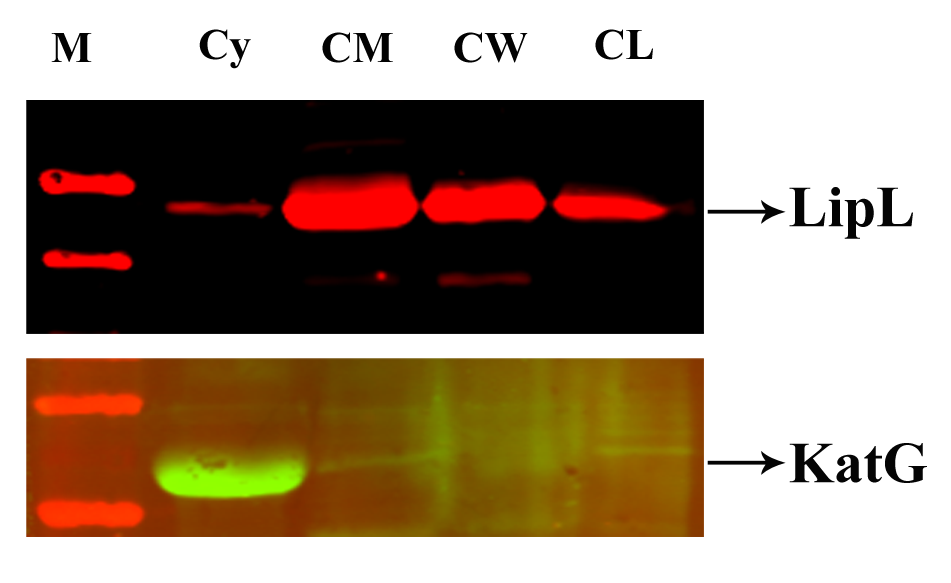

Supplement: S2 Fig — Bacteria were lysed and fractionated to separate the cytoplasm (Cy) from the cell wall (CW). Equal amounts of protein (20 μg) from each fraction were subjected to SDS-PAGE, transferred onto a nitrocellulose membrane, and probed with either monoclonal anti-His antibodies (top) or rabbit anti-KatG antiserum (bottom). (TIF) [file pone.0138151.s003.tif]

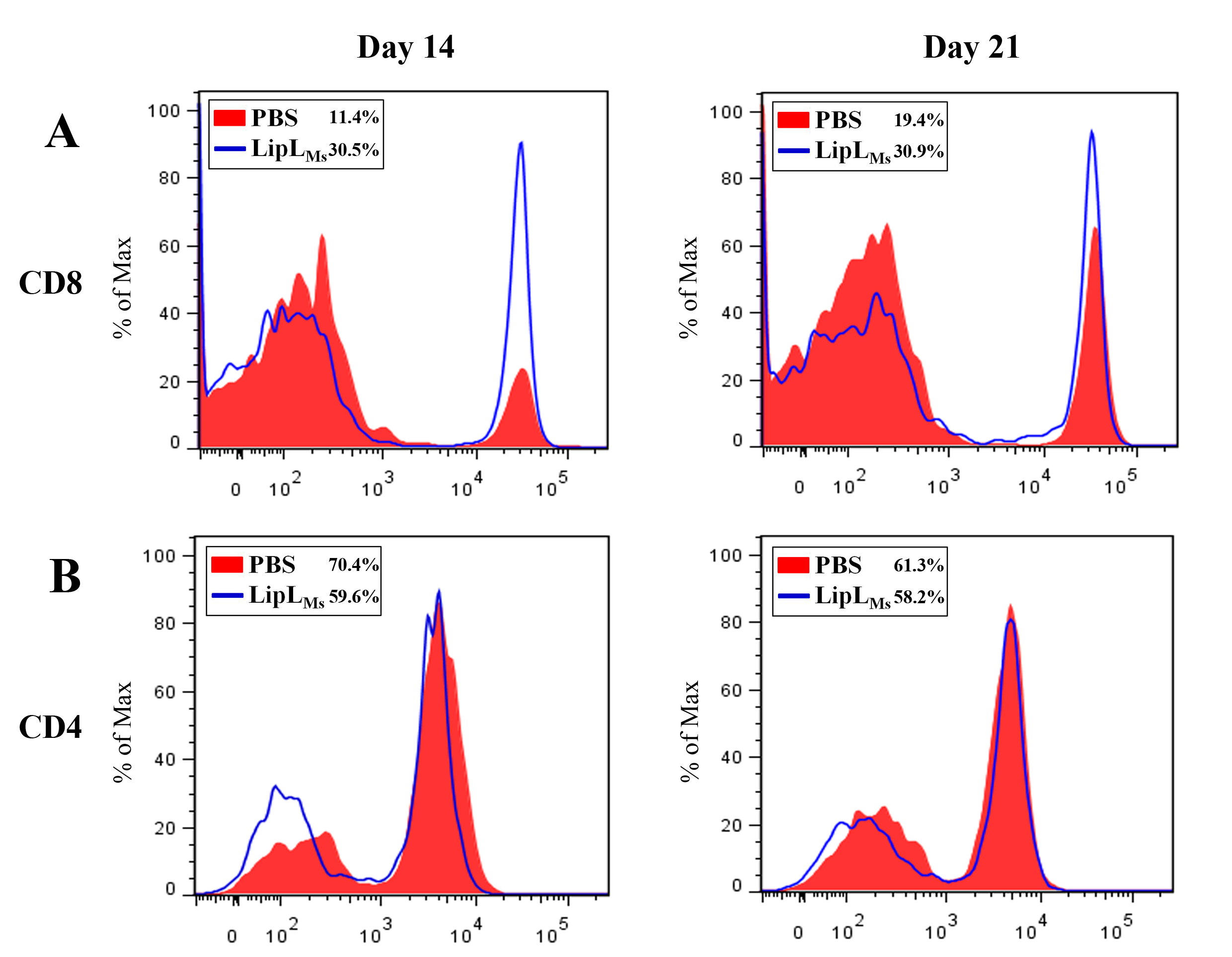

Supplement: S3 Fig — Histograms showed the CD3+ CD8+ T cell subsets and the CD3+ CD4+ T cell subsets from LipLMs and PBS immunized mice. (A) The percentage of CD3+ CD8+ T cells in the LipLMs group was significantly higher than that of the control group. (B) The percentage of CD3+ CD4+ T cells in the LipLMs group was significantly decreased compared with the control group (day 21 not significant). (TIF) [file pone.0138151.s004.tif]

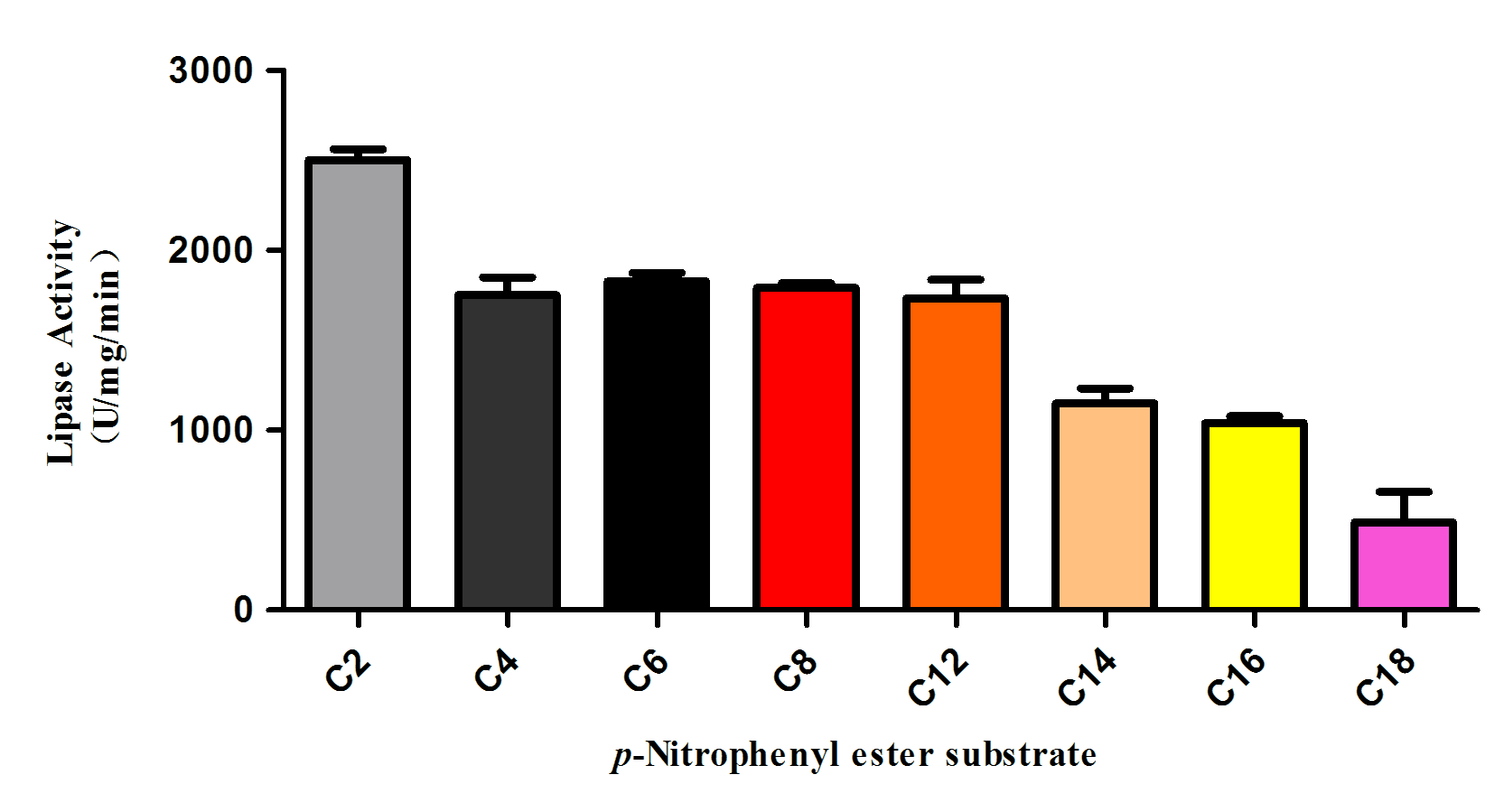

Supplement: S4 Fig — Lipase activity of LipY towards p-NP esters with various chain lengths (C2, acetate; C4, butyrate; C6, caproate; C8, caprylate; C12, laurate; C14, myristate; C16, palmitate; and C18, stearate). The values represent the means ± SD of three independent experiments. (TIF) [file pone.0138151.s005.tif]
